# Youth’s mental health journeys through the COVID-19 pandemic using arts-based methods in virtual environments

**DOI:** 10.1371/journal.pone.0349860

**Published:** 2026-06-05

**Authors:** Roberta L. Woodgate, Lisa Mary-Quigley, Ashley Bell, Marlyn Bennett, Andrew R. Hatala

**Affiliations:** 1 College of Nursing, Rady Faculty of Health Sciences, University of Manitoba, Winnipeg, Manitoba, Canada; 2 Faculty of Social Work, Werklund School of Education, University of Calgary, Calgary, Alberta, Canada; 3 College of Community and Global Health, Rady Faculty of Health Sciences, University of Manitoba, Winnipeg, Manitoba, Canada; University of Saskatchewan, CANADA

## Abstract

Already facing a crisis, youth mental health and well-being has been dramatically affected by the COVID-19 pandemic. Recognizing this decline in youth mental health is expected to last well into the future, there is an imperative to examine youth’s experiences with their mental health journeys throughout with COVID-19 pandemic, which will play a critical role in shaping their future mental health trajectories. Utilizing guidelines developed for using arts-based methods in virtual environments, this interpretive description study aimed to gain deeper insight into youth’s mental health journeys throughout the pandemic. Using open-ended interviews supplemented with youth-directed arts-based methods (ecomaps, journey maps, and creating an art piece of their choosing), thematic analysis was used to identify themes arising from the textual and graphical data. 19 youth (14–24 years) from across Canada shared their journeys with mental health and well-being throughout the COVID-19 pandemic. Based on key time points, three themes were identified: 1) Entering COVID-19: Chaos, 2) During COVID-19: Introspection, and 3) After COVID-19: Personal Growth. Journeying through the COVID-19 pandemic, youth experienced changes in their mental health and well-being experiences. Recommendations provide points to consider in supporting youth mental health and the utilization of arts-based methods to support reflection and introspection.

## Introduction

Prior to the COVID-19 pandemic, youth mental health was already facing a crisis, with a decline being seen over the past 20 years [1]. Youth (15–30 years) had the lowest rates of good/excellent mental health and the highest rates of suicidal thoughts, compared to people in older age brackets [1], and worldwide, one in seven youth (10–19 years) had a mental health disorder [2]. The COVID-19 pandemic accelerated this decline, with young people having the highest reduction in mental health during the onset of the pandemic [1]. A meta-analysis by Wang et al. [3] identified that rates of depression and anxiety among youth significantly increased during the pandemic, compared to rates seen previously. Similarly, across studies, Canadian youth saw increases in anxiety and depression symptoms over this period [4–6]. This was more pronounced for female youth, younger youth [4,7], youth from lower income households [7], and youth with worse family relationships [6].

It is expected that the mental health fallout among youth from the COVID-19 pandemic will continue to persist well into the future [8,9]. High rates of mental health challenges have continued to persist throughout the pandemic, with one study finding a decrease in mental health among young people from before to six months after the initiation of a COVID-19 vaccination campaign [7]. Youth have also reported a lingering sense of anxiety since the COVID-19 pandemic [10]. Youth with poor mental health can experience future challenges, including financial challenges and decreased income [11,12], as well as lower educational attainment and interpersonal relationship challenges [11].

Emerging adulthood, as defined by Arnett [13], is the developmental period of late teens to early twenties. Central to this stage is an increase in independence and a focus on the self. This time is integral to identity exploration, and there can be feelings of instability and transition uncertainty [13]. The COVID-19 pandemic resulted in significant interruptions to this expected development [14]. Widespread closures and social distancing measures affected youth’s daily lives, with alterations and stressors arising in their schooling, activities, and friendships. Routines were disrupted [14], and youth missed out on “rites of passage” and milestone events, such as graduations [15]. Youth experienced feelings of loss and longing attributed to missing out on the activities they expected to be doing at their age [16]. Emerging adulthood is also a critical point for the development of mental health conditions [17], with 75% of adult mental health disorders arising during this time period or prior to [18,19].

Negative impacts to youth mental health have been attributed to the pandemic’s public health measures/lockdowns, the loss of social activities, the transition to online/remote education, and challenges with familial relationships [20]. In one study, over 80% of young people reported that the COVID-19 pandemic had a negative impact on their work/school, mental health, and non-work/school life [14]. The disruptions in school, including a switch to remote education, was a notable stressor among youth, who struggled with uncertain expectations and a lack of support and communication from educators [15]. As a result, youth found it challenging to keep up with schoolwork [21]. Some youth also experienced tensions with their family, particularly when restrictions resulted in them living and working in the same space [15]. The pandemic also contributed to feelings of worry and uncertainty. Concerns about health and safety weighed heavily on youth’s minds [10]. Youth were also concerned for their future, with the pandemic’s uncertainty impacting their goals [14]. Youth felt an increase in fatigue, had difficulties sleeping, and were less physically active during the COVID-19 pandemic [21].

A profound contributor to mental health challenges during the pandemic for youth was the experience of social isolation and feelings of loneliness [10,15,16], as the pandemic saw a decrease in social interactions [21] and youth were unable to see friends and family outside of their home [14]. In one study, the most commonly reported stressor by youth during the pandemic was social stress, which resulted from limited time with friends and/or family, decreased social activity, and having plans change [22]. This was amplified for youth who lived alone [16]. As it was challenging for youth to meet new people, a decline in forming new connections was also seen [21]. Youth were stressed about this inability to meet new people and form relationships, as this is a developmental expectation for youth at this life stage [16]. Many youth reported that connecting with others primarily through technology made it challenging to form and maintain meaningful connection [14]. For others, technology supported social connection and helped to alleviate some of the mental health impacts of social isolation [10,16]. However, some youth noted the benefits of technology on social connection were only temporary, with loneliness returning after the online interaction [21].

To get through the challenges of the COVID-19 pandemic, youth utilized a variety of coping strategies. Experiencing social connectedness through supportive family, friends, and living environments was key to mitigating some feelings of loneliness [10,21,23]. Having an outlet to express and validate their emotions also helped youth cope [10,20]. Emotional well-being during the pandemic was associated with self-compassion and having psychological needs met at school [24]. Outside of this, engaging in hobbies also helped youth cope [20]. In one study, secondary control coping strategies were the most common ones used by youth during the pandemic; these are strategies that aim to regulate the response to a stressor, and include acceptance, distraction, positive thinking, and cognitive restructuring [22].

Though much of the literature tends to focus on the negative impacts of the COVID-19 pandemic on youth, a few positive impacts have been seen. For some youth, the ability to slow down at the onset of the pandemic and associated lockdowns had a benefit to their mental health [15]. This reduced stress and improved well-being, particularly for youth who had very busy lives before the pandemic [21]. As well, some youth felt relief to no longer have to attend social events that they previously would have been pressured to do [21]. Youth have also highlighted that they experienced personal growth over the pandemic, as they had time and space to build on their individual capacity, focus on themselves, and engage in self-reflection [10,14,21]. Additionally, youth found benefits to having time to pursue personal hobbies and activities [15]. Some also found that they were able to create closer bonds and relationships with the people they lived with over the pandemic [14,15,21].

Arts-based methods (ABM) may provide a unique means to understanding youth’s experiences and mental health during the COVID-19 pandemic [25]. ABM can include a variety of mediums, such as, but not limited to, photovoice/photography, drawing, painting, poetry, and performance arts, such as dance or theatre [26,27]. Integrating ABM into qualitative research can support the sharing of sensitive thoughts and experiences, as well as provide enhanced nuance to such experiences [25]. ABM help youth express themselves and their experiences when words themselves may be difficult, providing richness and depth to the experiences shared [28–30]. It can also serve as an emotional outlet and a means to self-reflect on experiences for youth, providing therapeutic benefits to participants [28–30]. ABM further aid in reducing power imbalances seen between researchers and participating youth [29]. As a participatory research method, ABM allow active engagement and collaboration with youth throughout the research cycle, which strengthens knowledge translation [26,27,29,31].

ABM can support obtaining a more complete picture into how youth themselves experienced transitions during the COVID-19 pandemic, and the impact of this on their mental health and well-being. However, in light of social gathering restrictions placed as a result of public health measures during the COVID-19 pandemic, research using ABM had to transition to online modalities. This presented unique challenges, given its’ reliance on a collaborative relationship between participants and researcher [25,32]. To support youth mental health research with ABM using online approaches, guidelines co-designed with youth were developed by Woodgate et al. [25]. This study aimed to implement and examine these new guidelines for using ABM in virtual environments in a qualitative arts-based study exploring youth’s reflection on their mental health and well-being experiences throughout the COVID-19 pandemic. For the purposes of this study, Public Health Agency of Canada’s definition of mental health and well-being was used, which is “the capacity of each and all of us to feel, think, act in ways that enhance our ability to enjoy life and deal with the challenges we face. It is a positive sense of emotional and spiritual well-being that respects the importance of culture, equity, social justice, interconnections and personal dignity” [33].

## Methods

### Research design and framework

This arts-based, qualitative study was guided by the methodology of interpretive description which acknowledges the constructed and contextual elements of human experience to identify themes with the aim of creating an interpretive description capable of offering practical solutions to inform practice, policy, and research [34,35]. Woodgate et al.’s guidelines for using ABM in virtual environments, which was co-designed by youth [25], was also used. With the overarching principle of sustaining mindful presence in research, key tenants of using ABM in virtual environments include creating a safe space, ensuring youth have a say, facilitating meaningful engagement, and paying receptive attention throughout the research process (see Table 1) [25]. These aspects were applied at all stages of the research process. As well, as part of this study’s commitment to youth-centered and -directed research, 7 youth co-researchers were actively involved throughout the study. This study further utilized Woodgate’s Youth Engagement in Research Framework to ensure youth participants and co-researchers were meaningfully engaged throughout, resulting in more inclusive and culturally responsive research [36,37].

**Table 1:** Key principles of using ABM in virtual environments with youth.

Principle	Characteristics
(1) Creating a safe space	Provide accommodations Let youth self-identify Incorporate diversity & inclusion Consider language (e.g., gender-inclusive language, plain language, provide translation if needed). Be mindful of ethical considerations
(2) Youth having a say	Youth have a choice in type of ABM to use Youth have a choice in complementary research methods used for the research (e.g., interviews, focus groups, breakout sessions)
(3) Facilitating meaningful engagement	Use virtual platform that is most comfortable for youth (e.g., Zoom, Facetime, etc.) Have options & flexibility in ways to communicate during research activities (e.g., chat function, microphone, etc.) Provide option & flexibility for research participation at times outside of the research activities (e.g., group chat, email group, etc.)
(4) Paying receptive attention throughout the research process	Identify & address barriers in accessing and using technology Offer ABM training for youth Provide necessary resources and tools for creating art Be mindful of technology fatigue & burnout Provide recognition & reward

While the methodological approach employed in this study was carefully designed to align with the principles of arts-based research, it is important to acknowledge that the process was not without its challenges. For example, participants’ time constraints and feelings of insecurity in their ability to create art. These challenges, which required constant negotiation, shaped the trajectory of the research. The guidelines developed for using ABM in virtual environments (see Table 1) provided direction to address these challenges. Nonetheless, a more detailed exploration of these challenges will be addressed in a separate paper, and future research is warranted to investigate if these guidelines need any revisions or additions to support youth’s full and active participation.

### Recruitment and data collection

Eligible participants were youth, defined as young people aged 14–24 years, who resided in Canada. Participant recruitment and data collection took place between May 1, 2023, and May 10, 2024. Participants were recruited using convenience sampling through targeted social media recruitment (e.g., Instagram), as well as advertising at youth-serving organizations. Once a youth expressed interest in participating in the study, they were provided with additional information about the study and informed consent was obtained. They were invited to participate in two open-ended interviews supplemented with the arts-based activities of ecomaps, journey maps, and the creation of an art piece of their choosing. Interview guides were developed in collaboration with the youth co-researchers. Prompts for the arts-based activities and the interviews asked youth to reflect on their experiences through the COVID-19 pandemic, with attention to their mental health and well-being.

Prior to their first interview, youth were instructed to develop two ecomaps. Ecomaps are a graphic portrayal of individual’s social relationships and networks, and they may include important events, places, or other aspects of youth’s lives [38,39]. Youth were instructed to draw circles that represented the most important aspects of their lives, including their relationships with their friends and family, activities they liked to do, and places they liked to visit. Youth then drew different types of lines between the circles to indicate the degree of connection they have with each person, activity, or place. For example, a thicker line for a stronger connection or a wavy line for a tumultuous connection. The first ecomap detailed the important factors in youth’s lives pertaining to their COVID-19 experience. The second ecomap detailed important factors affecting youth’s lives in the present day, after the pandemic. With these two ecomaps prepared, youth then took part in their first open-ended interview, which guided them through a series of questions and prompts aimed at eliciting their current and past experiences with the pandemic, including the impact on their mental health and well-being.

Prior to their second interview, youth were provided with instructions on completing a journey map of their experience with the COVID-19 pandemic to show a visual depiction of its impact on their mental health and well-being. Journey maps are graphical depictions of one’s lived experience [40,41], which helps to understand their experiences as they navigate complex and dynamic systems [42,43]. Journey maps include both time and emotional response dimensions, as well as words, drawings, and symbols to provide a visual narrative timeline of an experience [44]. Common elements of journey maps are touchpoints or milestones at pre-, during- and post-experience; in this case, the COVID-19 pandemic [45]. In creating their journey map, youth were provided with guidelines developed by Woodgate et al. [46] Youth were also asked to consider how their mental health changed throughout the different stages of the pandemic, as well as the factors that influenced these changes.

In addition, youth were tasked with creating an art piece of their choosing to help demonstrate their experience with the COVID-19 pandemic and the impact of this on their mental health and well-being. These art pieces could be of any medium of their choosing, for example, photos, drawings, poetry, or dance [25]. They were provided with a workbook that provided instructions and guidance for this activity. It included information on the research study and what arts-based methods are, examples and details of possible mediums youth could choose, prompts to consider when creating their art piece, the research team’s contact information, and available mental health resources, if needed. Following the completion of their journey map and art piece, youth then participated in their second interview, in which youth discussed their artwork, including how it was created and the meanings behind it.

Throughout the arts-based activities, a research coordinator checked in with all participants to address any questions or concerns that arose during their art creation process. The research coordinator also asked youth about the medium of their chosen art piece, and inquired and offered assistance for youth who needed additional art supplies to carry out their vision. Youth were also provided with the research coordinator’s contact information if they had further questions. To minimize feelings of intimidation or anxiety when participating in the arts-based activities, it was stressed to youth in their instruction that the arts-based activities were to be used to help them reflect on their personal experiences [47], and not as a judgment of their artistic abilities.

All interviews took place virtually over Zoom. They were digitally recorded on Zoom, with the video file deleted and only the audio file saved to protect participant confidentiality. These audio files were later transcribed verbatim to preserve authenticity. Field notes were recorded after each interview to describe nonverbal behaviours, interviewer-interviewee dynamics, and additional context.

### Data analysis

Data analysis occurred concurrently with data collection, and the inductive qualitative analytical approach of interpretive description was used. By simultaneously collecting and analyzing data, any inconsistencies or new insights were further examined through the ongoing interviews.

Qualitative data analysis included textual data (interviews, fieldnotes, words/statements from the arts-based activities) and graphical data (drawings/symbols from the arts-based activities). The graphical data served as visual representations of the textual data, helping to inform the themes emerging from the youth’s data and provide a deeper understanding of their perspectives and experiences. Interview field notes were shared with the research team to provide additional details of the interview context and the interviewer’s reflexive account. The research team was fully immersed in the data by reading and re-reading transcripts, looking for significant statements that addressed the study’s aim of learning about youth’s mental health journeys throughout the COVID-19 pandemic. Open codes were discussed, collated, and examined for significant broader patterns of meaning (potential themes). This involved identifying recurrent themes across participants and data sources, as well as delineating units of meaning from the data, clustering units of meaning to form thematic statements, and extracting themes [48–50], which were organized into a table of contents in Microsoft Word. The first 10% of the raw data was analyzed independently by the authors, allowing discussion of the emerging themes until agreement was achieved. This theme list was then applied to the remaining data and inter-coder reliability was calculated to determine the percentage agreement and Kappa coefficient. Once sufficient, analysis was then completed using the established theme list. The research team was engaged in frequent discussions regarding the emerging themes until a consensus was reached on the final list. Any disagreements in the thematic analysis were brought up to the larger research team and debated until agreement was reached on how to proceed. Throughout the analysis process, team members engaged in reflection as individuals and as a team to help ensure open-mindedness was maintained, and to reduce personal biases from impacting the analysis.

Graphical data, including the ecomaps, journey maps, and other arts-based activities, were used to supplement and complement the corresponding textual data by looking at similarities, differences, and absences between both forms of data [51]. During analysis, the coded textual data was revisited with the related graphical data by asking the following questions: 1) What is in the visual representations (i.e., drawings/symbols) that supports or reinforces what was learned from the textual data?, 2) What is in the images that challenges what was learned from the textual data?, 3) What is in the images that is not in the interviews?, and 4) What is in the interviews that is not in the images? [51]. These questions helped to facilitate comparison between the textual and graphical data sources, considering the possibilities for congruence or divergence [51]. Notes and memos were made to identify and examine overlaps between these data sources, assessing whether they complemented, challenged, or revealed different aspects of youth’s experiences. Themes were further refined through this process. The integration of textual and graphical data helped to enhance the depth and clarity of analysis by affording a visualization of youth’s experiences, while grounding interpretations in their direct quotes and narratives. The integration also helped to identify patterns, inconsistencies, and unique insights that might not be apparent when each data type is analyzed in isolation.

### Ethical considerations

All methods were performed in accordance with the Declaration of Helsinki. Ethics approval was obtained through the University of Manitoba’s Research Ethics Board 1 (Protocol # HE2022−0101). All participants provided free and informed written and verbal consent to be recorded, and that quotes, ecomaps, journey maps, and artwork be used in publications. All youth participants were made aware that any publications resulting from the study would not include identifying information. Confidentiality and anonymity were ensured by assigning to each participant a code which was used to identify the interview transcripts. Youth were also provided with an honorarium for each interview they participated in and for the art piece creation process.

### Participants

All participants filled out a demographics form prior to their first interview. Overall, 19 youth from across Canada participated in this study (see Table 2). Youth ranged in age from 14–24 years (M = 20.6 years), and included 12 female youth, 6 male youth, and 1 non-binary youth. The majority of participants were racialized youth, worked for pay, and did volunteer work. Participants were diverse and had diverse life experiences. They had different family structures and living arrangements. Some were students, some were working, and others were looking for work. Experiences with mental health challenges varied, from having diagnosed/self-diagnosed mental health conditions, to no diagnoses. All participant names reported are pseudonyms.

**Table 2:** Participant characteristics (N = 19).

Variable	Number
Gender	
Female	12
Male	6
Non-Binary (Two-Spirit)	1
Age	
14	2
16	2
19	2
20	1
22	5
23	4
24	3
Ethnicity	
South Asian	8
East Asian	2
Caucasian	2
Black	2
Mixed	2
Southeast Asian	1
Indigenous (Metis)	1
Prefer not to answer	1
Sexual Orientation	
Straight/Heterosexual	12
Prefer Not To Answer	2
Bisexual and Demisexual	1
Bisexual and Questioning/Unsure	1
Demisexual	1
Questioning/Unsure	1
Asexual/Aromantic	1
Education	
University Degree	8
Some University	2
Some College	3
High School Diploma	2
Some High School	2
Grade 8 or less	2
City of Residence	
Winnipeg	12
Toronto	2
Steinbach	1
Edmonton	1
Calgary	1
Vancouver	1
Montreal	1
Do You Work For Pay?	
Yes	10
No	9
Do You Volunteer?	
Yes	10
No	9

### Findings

Throughout their journey across the COVID-19 pandemic, youth described key time points that shaped their mental health experiences, which formed the basis for the identified themes: (1) Entering COVID-19: Chaos, (2) During COVID-19: Introspection, and (3) After COVID-19: Personal Growth. These stages were not necessarily linear; for some youth, phases were re-entered as the pandemic and various restrictions changed. The time spent in each phase also varied among participants, as well as their definitions of when each stage began.

### Entering COVID-19: Chaos

**Fig 1:**
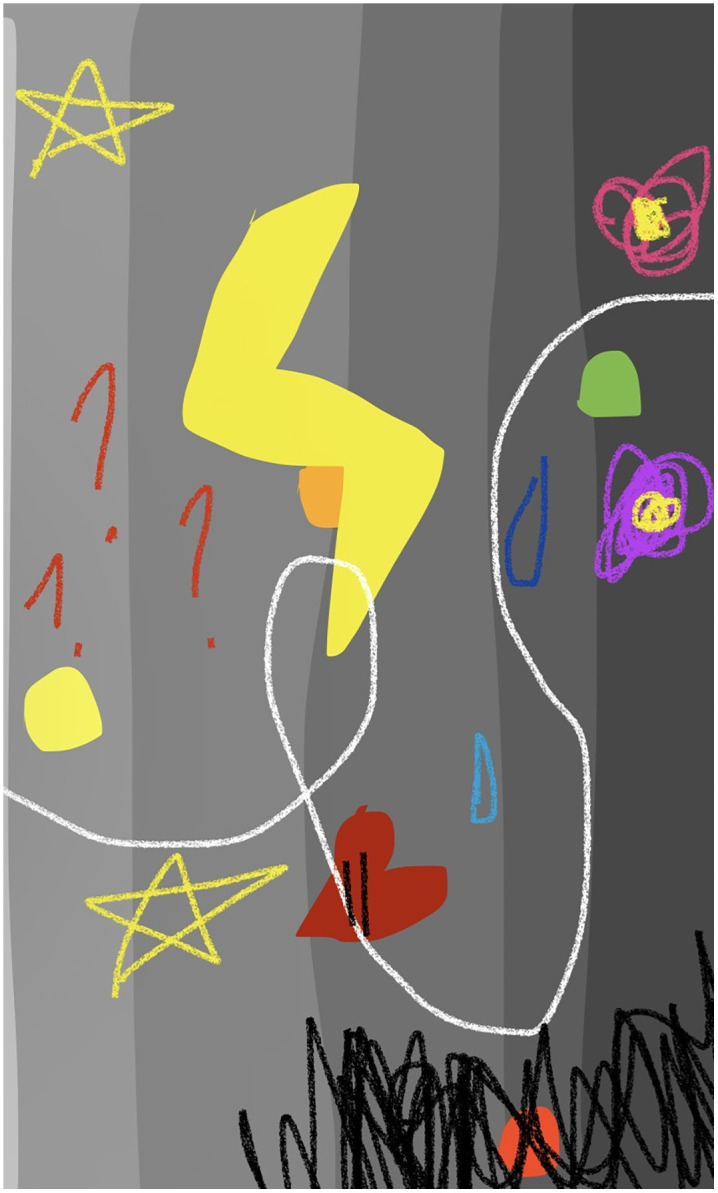
Hana’s journey map.

Participants experienced feelings of chaos during the initial phases of COVID-19. As Jule shared, “It was really chaotic” (Jule, 23 years), while Sienna identified her introduction to COVID-19 as “a time of chaos and unknown” (Sienna, 20 years). As the pandemic neared, Hana created a rainbow of tortuous connected symbols in her journey map (see Fig 1) to demonstrate “the chaos of what’s going, what actually happened” (Hana, 17 years). This theme of chaos has been divided into three sub-themes: (1) Not Prepared, (2) Time Takes on New Meaning, and (3) Feelings of Doom and Gloom.

### Not prepared

Youth expressed that they were not prepared for the onset of the COVID-19 pandemic. As Kora shared, “I feel like nobody was really prepared for it” (Kora, 14 years). Callum also noted, “Nobody was prepared and they really had no idea what to do” (Callum, 24 years). Unpreparedness is further highlighted by Mina, who immigrated to Canada shortly before COVID-19 started. She commented, “I was definitely not prepared for that when we as a family chose to come here” (Mina, 16 years). Youth expressed that the start of the COVID-19 pandemic was a whirlwind of uncertainty. As Callum stated, “You know, it was new for all, you know, this pandemic thing, you know?” (Callum, 24 years).

In the beginning of COVID-19, youth also described perceiving previously familiar things as different or new. For example, Cali described how the arrival of COVID-19 changed their perspective of home, “like what do I do now, like I’m put in a position where home doesn’t feel like home” (Cali, 23 years).

Participants voiced unawareness of needs and expectations, length of the pandemic, and understanding of feelings and emotions as COVID-19 neared. Cali recalled, “I didn’t know what choices to make and I think that is when you, metaphorically when you don’t know what choices to make it’s like you’re in chains ‘cause like you don’t know what way to go” (Cali, 23 years). Not knowing what to expect as COVID-19 neared also reflected the unawareness that was felt by youth. Hana commented, “COVID made like everybody feel helpless, like we didn’t know what was going to happen you know” (Hana, 17 years). Similarly, the uncertainty regarding the pandemic’s duration was an additional concern for youth. As Paige noted, “just being like, like wondering when it’s like going to be over” (Paige, 14 years), and Kora stated, “I feel like I was like, really stressed about like, when’s this going to end ‘cause I did not enjoy like a second of it” (Kora, 14 years).

Participants shared that the countless changes to their everyday lives made it difficult to adjust to their new reality at the start of the COVID-19 pandemic. Finn initially struggled with the transition to virtual education, commenting, “having switched to a different format [of schooling] completely all of a sudden, I felt like, the time to adapt wasn’t really there during that time” (Finn, 22 years). Amirah similarly offered, “it [the virtual environment] was really difficult to adjust to” (Amirah, 22 years). Feeling burdened by the number of adjustments that had to be made was noted by Jule, who recalled, “people were panicking, it was like oh we have to adapt to so many things” (Jule, 23 years).

### Time takes on new meaning

This sub-theme refers to nuanced patterns of time experienced by the youth participants as the COVID-19 pandemic began. Several participants identified the arrival of COVID-19 as a historic and unprecedented experience across the world. This acknowledgment of a shared, novel connection is evident in statements made by Kora and Paige, who shared, “it was like a first-time experience for everybody, obviously” (Kora, 14 years), and “it was like kind of weird that like, it was happening to everybody but in different ways” (Paige, 14 years). The sudden onset of the pandemic was also specifically jarring for youth. As one stated, “when it shifted directly from in-person to online, it was very sudden” (Finn, 22 years).

For other youth, time felt like it paused at the onset of the pandemic. For Jule, this pause allowed her to slow down and rest, as she stated, “Yeah it [COVID] finally sort of gave me a break… COVID happened and it’s like everything came to a stop” (Jule, 23 years). Pauses were also used by youth to describe taking time for enjoyment and breaks from normal routines. Viewing a pause as a welcomed event, Lexi shared:

“When everything kind of locked down, all of a sudden it was like, just you couldn’t go anywhere, so you were stuck kind of at home, and it was kind of exciting to you know not have to go to school, not have to go to work. It was like a two-week vacation period.” (Lexi, 23 years)

This pause in time also impacted youth’s involvement in social activities and extracurriculars. This was exemplified by Rohan, who noted, “So my social life was school, clubs, anything to do with like extracurriculars was all school-based… So the second that the pandemic hit it was kind of like nothing” (Rohan, 19 years).

### Feelings of doom and gloom

At the outset of the pandemic, youth participants collectively offered negative feelings and emotions. Feelings of fear and anxiety were commonly highlighted. Amirah commented, “I go to school out of province from where I’m originally from, so I basically had to suddenly fly home… and leave and that was just terrifying” (Amirah, 22 years). Another participant identified these emotions as a younger person, recalling, “Yeah, I was definitely really scared” (Kora, 14 years). An additional participant recounted having feelings of anxiety, “At the beginning of COVID-19 my mind would just race” (Mei, 22 years).

Participants also commonly described feelings of sadness as the COVID-19 pandemic began. Hana explained the colours in her journey map (see Fig 1), noting, “it’s grey because it’s sad” (Hana, 17 years). Sadness was described by Mina as well, who recalled, “one thing that I remember the most about that period is that I spent most of my days in the toilet. ‘Cause that was the only door that could be locked. And so, I was like when I’m sad I just go in and like kind of like cry a bit” (Mina, 14 years).

The onset of the COVID-19 pandemic also brought experiences of loneliness for youth. Paige discussed this, stating, “So I just watched a lot of TV and like did a lot of stuff by myself which it, it was sometimes like pretty lonely and everything and weird” (Paige, 14 years). A similar expression of loneliness during the onset of COVID-19 was shared by Rohan, “I think that feeling, feeling that I’m lonely and knowing that I didn’t really have that many people to reach out to at one point… It definitely did affect me” (Rohan, 19 years). Rohan also recalled how he retreated from his social circle:

“It was in the beginning… I was just going through a little period of just I just wanted to be alone. I pushed like a lot of my friends away, I stopped talking to a lot of my peers, and then I deleted social media, and I basically threw away my phone, I hid it in the house and like I completely forgot where I left my phone.” (Rohan, 19 years)

### During COVID-19: Introspection

**Fig 2:**
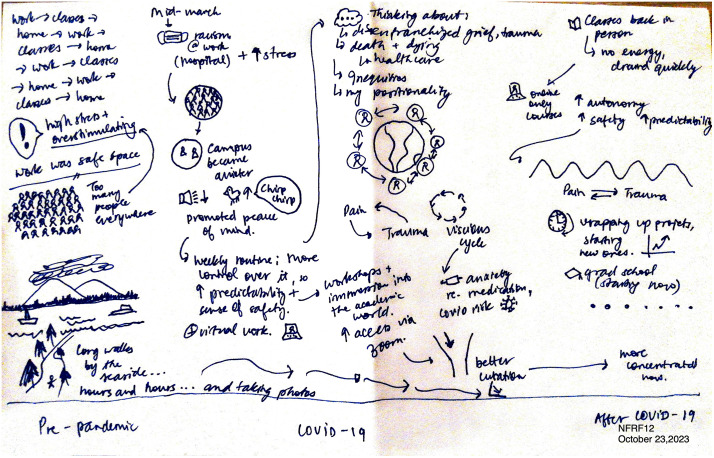
Jule’s journey map.

As the COVID-19 pandemic continued, youth began to engage in a journey of introspection by looking inward to learn about their mental health and well-being. Finding themselves in less hurried environments and having more free time during the pandemic prompted many youth to slow down and check in with themselves. As Jule demonstrated in her journey map (see Fig 2), during the COVID-19 pandemic, she took time to think about mental health and her position in the world, with each word, phrase, and image capturing the various thoughts and reflections she experienced. She explained, “I got an opportunity to sort of, I guess, metabolize my own grief experience” (Jule, 23 years). Sub-themes within introspection include: (1) Accepting their COVID-19 reality, (2) Coping, and (3) Emotional toll.

### Accepting their COVID-19 reality

As the pandemic progressed, youth gradually began to accept this as their reality. Through introspection, the significant impact of the pandemic upon their lives was understood and accepted for what it was. In discussing her life during COVID-19, Paige identified that aspects of the pandemic (e.g., masking and social distancing), “kind of became like part of your life almost” (Paige, 14 years). She also described the pandemic as an experience, stating, “I feel like I don’t really think of it so much as like a virus, I kind of just thought of it as like an experience almost… it was just like something that was like there that we had to like get through” (Paige, 14 years).

For others, they felt a loss related to pandemic changes. This is revealed by Sienna, who experienced a loss of control, stating, “Yeah, like very like loss of control in a sense too… Like you’re being, you’re being overcontrolled. But you feel like you have a loss of control ‘cause some other bigger power” (Sienna, 20 years). A loss of time was echoed by a couple of participants. Hana stated:

“It kind of feels like my life has changed as a result of COVID because it’s just like, I don’t know, the fear of it happening again and I still feel like I am, like I’m 15 but I’m actually 17. It feels like I just like lost a bunch of time in-between that.” (Hana, 17 years)

Likewise, Mei shared, “COVID kind of took two, two and a half years out of my life and like stopped it…. And sort of paused two, two and a half years and sort of like I don’t know in a way like took a chunk out of my whole life” (Mei, 22 years).

COVID-19 also changed several participants’ plans for their futures. For Ruvi, this was seen in a delay in immigration to Canada to begin post-secondary education, which “hampered my everything. My study plan, my time, my years. Everything” (Ruvi, 22 years). For Paige, this interrupted a critical moment in her professional ballet training, which, ultimately, ended her future career in ballet:

“Me and my friends, like, we had all of our first solos and everything that we were going to go to competitions with and we were doing like a big festival and then COVID happened, so we had to stop like before we even got the chance to perform, like I think it was the day of.” (Paige, 14 years)

**Fig 3:**
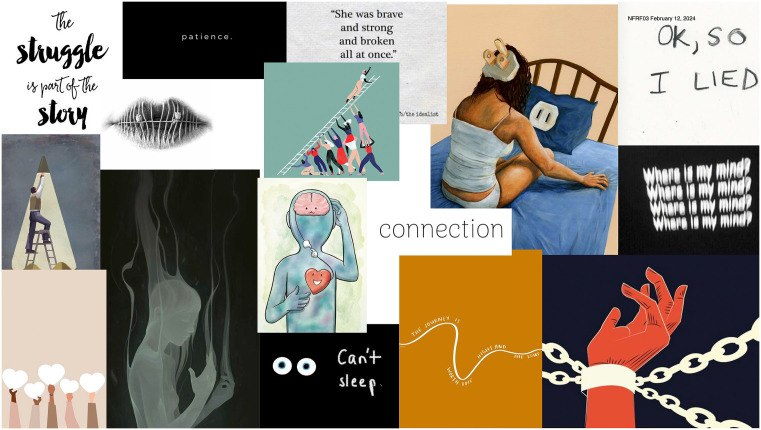
Cali’s digital mood board.

When sharing their individual situations during COVID-19, the youth described an internal strain when participating in introspective activity. For Cali, this struggle was an integral part of her introspective process, as illustrated in Fig 3. She stated: “So the first thing is like the struggle is part of the story, hundred percent agree. If you talk to me now and you say what the story was, I would be like yeah the struggle was a part of the story” (Cali, 23 years).

For other participants, changes from the pandemic were viewed positively. Jule, who was more introverted, stated, “I think overall it was just brought out the hermit in me I think, which is great… it was perfect” (Jule, 23 years). Yuki felt freedom attributed to the COVID-19 pandemic, stating, “it’s just the feeling of I can stay at home, I can do whatever I want. I don’t need to go out, like I have an excuse to not be forced to go out… And you know do the normal things with the family, so I felt a bit free” (Yuki, 19 years).

### Coping

Several youth described engaging in introspection to help cope with the reality of the pandemic. Jule explained that the pandemic was “the first time [she] got the headspace to process a lot of things” (Jule, 23 years). This was also explained by Paige, who stated:

“I think it was really like being able to slowdown and like not having to go to work any, anymore, or not having school for like the like two weeks where everything was just locked down and it was kind of like, okay I have the time to just like check-in on myself.” (Paige, 23 years)

Youth coping strategies during the pandemic were unique to the individual, relying on methods that were tailored to their needs. Introspection allowed them to find the coping strategies that worked best for them. This is explained by Ellis, who shared, “So I think it [COVID] almost forced me in a way to figure out what works best for me” (Ellis, 23 years). The importance of tailoring a plan to the specific individual is also demonstrated by Lexi, who believed it was important to “just like explore and see what I have been doing, what I haven’t been doing, where I could make those improvements” (Lexi, 23 years).

### Emotional toll

Through introspection, youth were able to engage with their emotions and recognize their thoughts and feelings that were arising during the pandemic. Younger participants shared feelings of unpreparedness managing their emotions during COVID-19. As explained by Mina, “And for me it was like my puberty and like changing, like physically changing, mentally changing and I wasn’t like understanding who I am” (Mina, 16 years). Another younger participant, Rohan, described how life inexperience prior to the pandemic affected him, “like I was a kid at the time and I still am like very young… But with that mindset of like I haven’t really developed like strong attributes at that time, it definitely did affect my mind, it definitely did have adverse effects” (Rohan, 19 years).

**Fig 4:**
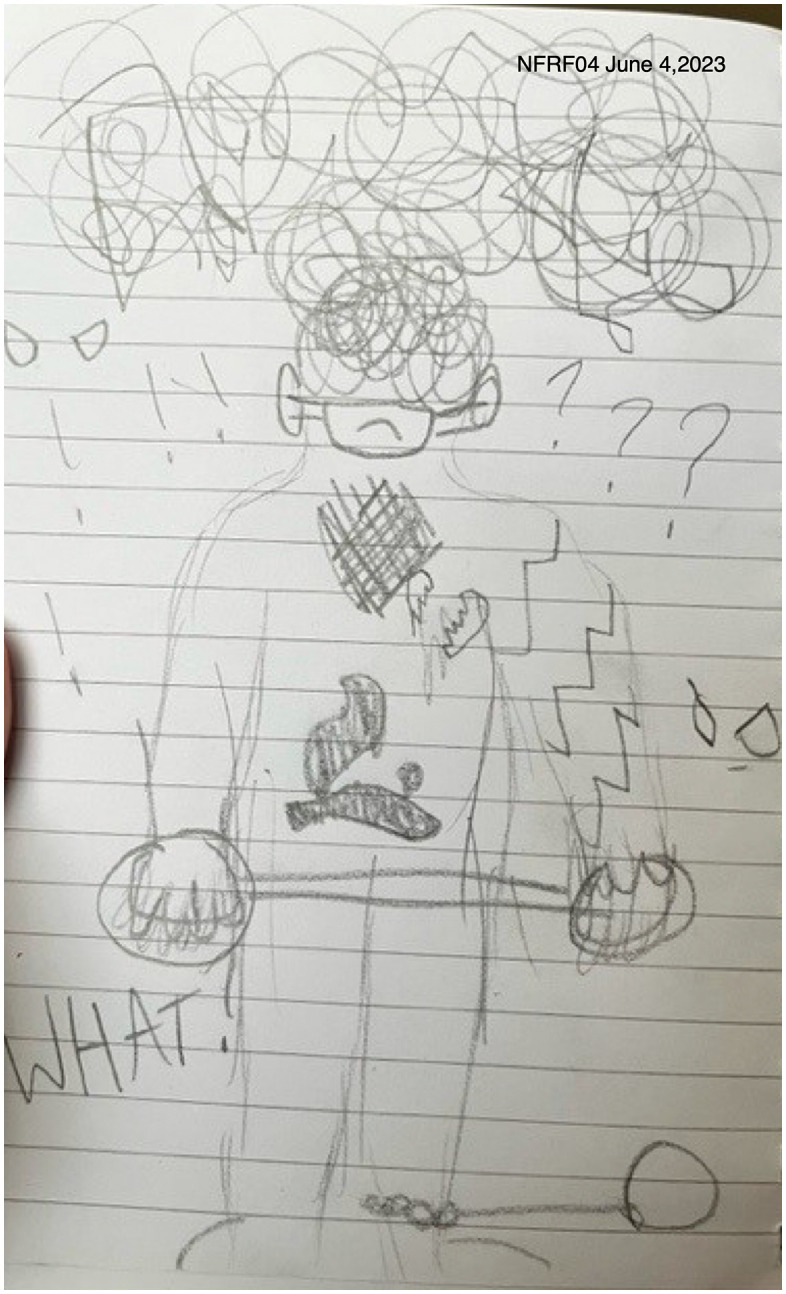
Hana’s pencil drawing.

Participants with diagnosed or self-diagnosed mental and/or physical conditions appeared to have poorer management of their emotions and feelings during COVID-19 in comparison to those who did not. When explaining a picture (see Fig 4) she created for this study, Hana, who had physical and mental health challenges, described the emotional toll she felt during the pandemic. She stated, “it’s just trying to like represent like feeling like just chained down and like, like you’re, you’re just, I just felt like it’s another representation of how like COVID made like everybody feel helpless” (Hana, 17 years). Overall, for her, the chains in the picture symbolized the feeling of being trapped and helpless during the COVID-19 pandemic, unable to do anything or know what would come next.

### After COVID-19: Personal growth

As the urgency of the COVID-19 pandemic began to diminish, personal growth was identified among the youth. Personal growth refers to the important lessons participants learned throughout the pandemic and aimed to continue practicing in the post-pandemic era. This theme is further divided into five sub-themes including: (1) I Can Do It, (2) Gratitude, (3) It’s OK to Talk About It, (4) Lessons in Action, and (5) Art as a Window to the Past.

### I can do it

This sub-theme refers to the development and strengthening of confidence among youth participants as the pandemic came to an end. Sharing how her confidence had grown, Cali explained: “I actually found those opportunities, but I had to do them solo... I did that all by myself, so I was really proud of that… my confidence got better” (Cali, 23 years).

**Fig 5:**
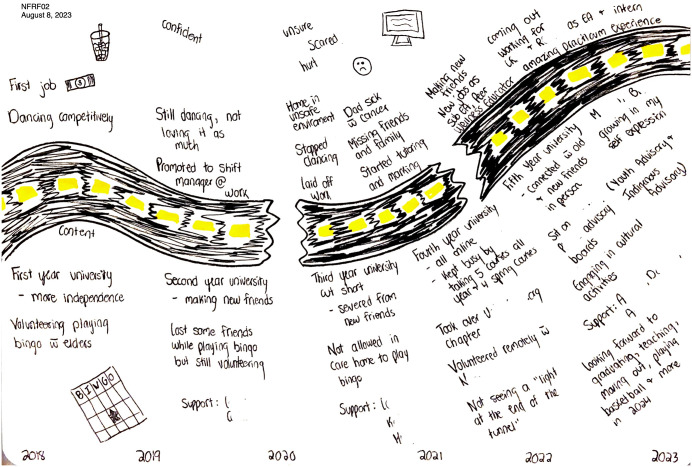
Ellis’s journey map.

Several youth appeared to have greater confidence in their ability to advocate for themselves in comparison to before the pandemic. For Ellis, their journey map showed this growth in their self-confidence over the pandemic (see Fig 5), noting near the end that they were “growing in [their] self expression” (Ellis, 23 years). They also highlighted this growth in themselves during their interview, explaining, “now I’m better at saying ‘no, you can’t treat me like that’ and I will say that, whereas before I wouldn’t and I would just kind of let everyone step over me where now I’m just like no, no” (Ellis, 23 years). Cali similarly identified that she no longer fears speaking up for herself, “I didn’t speak up for myself a lot pre-pandemic… That changed, that really changed… at the end now like after the pandemic a hundred percent that has changed. That I won’t let anyone say anything to me anymore” (Cali, 23 years).

For Jule, she became more confident in herself and her abilities. She stated:

“Going back to the growth piece, I think it sort of comes back to the realization that I can create these spaces myself and I don’t have to depend on other people who somehow naturally let go of their instincts and create that for me.” (Jule, 23 years)

Participants additionally described enhanced confidence in their decision-making skills following the COVID-19 pandemic, based on an enhanced understanding of what was best for them. Yuki shared his heightened self-confidence post-pandemic:

“I feel very different, I feel more organized, I feel like I know what I want to do. I don’t feel as passive because back then I was just passive just, just following the schedule that the school set for me, now I can feel like I have more authority over myself, I can go out, I can choose to go out, I can choose to make friends. I can choose how to schedule my day.” (Yuki, 19 years)

On the contrary, overconfidence was also identified as an issue by a couple of participants. For Theo, this overconfidence resulted in delaying mental health care. He stated, “even when it got worse I was still resilience and still trying to you know fight against the depression… it caused me not to try to reach out, I think I maybe got too comfortable in that space of trying to be resilient” (Theo, 22 years).

### Gratitude

Gratitude is the acknowledgment and feelings of thankfulness that the youth participants developed during the COVID-19 pandemic. This was commonly described by the participants when reflecting on their outcomes of the COVID-19 pandemic, specifically regarding the maintenance of their own health and the health of their family members. Imari reflected, “We realize the importance of life and we survived that situation” (Imari, 24 years). Another realization of gratitude was expressed by a Sienna, who stated, “like you don’t realize all the things you take for granted” (Sienna, 20 years).

Gratitude for social connection between family and friends emerged when reflecting back on COVID-19. Kora explained, “I feel like I started kind of appreciating like the time of COVID to like spend with my family or something” (Kora, 14 years). This was also stated by Paige, who recalled:

“It just reminds me of like the best things about COVID I feel like is because you didn’t really have that much freedom to like go and meet new people and everything, you kind of became really close to the people that you like had. So, for me, they were like some of my best friends and it was nice to just have those people.” (Paige, 16 years)

In response to COVID-19, several of the participants revealed gratitude for their experiences and realizations in their own unique journeys during the pandemic. As put by Lexi:

“I guess just like appreciating like the COVID process and seeing like what the lessons learned from that. So, like what can you, like can we take from that experience, like what did you learn during that it’s definitely an important process just to recognize.” (Lexi, 23 years)

**Fig 6:**
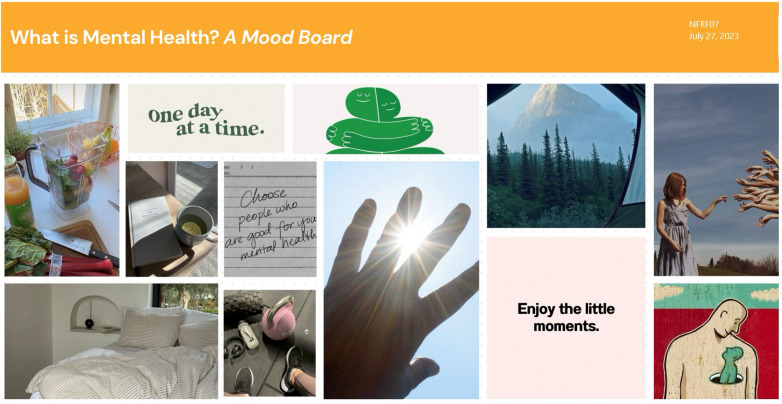
Lexi’s digital mood board.

She additionally indicated her development of gratitude which was a key feature in her mood board (see Figure 6):

“Enjoying the little moments. So, I think this comes back to some of like the gratitude that I’ve been doing, so feeling like the sunshine on my skin, hearing like the birds chirp in the morning, it’s things like that that make me, that I guess kind of ground me and make me realize that how lucky I am and how beautiful the world around me really is.” (Lexi, 23 years)

The development of appreciation during COVID-19 has also been demonstrated among youth participants who took on new initiatives during this period. Theo explained this appreciation:

“And I’m just appreciative, that’s why I’ve been going in my head, COVID was a blessing to me, because I’ve started so much things, or I got into so much things that I wouldn’t have gotten into if COVID didn’t happen.” (Theo, 22 years)

This was similarly recounted by Cali

“Going to these conferences, meeting people, very different for me. Sitting on a meeting, being able to talk to a professor and carrying that conversation on your own, that was different for me. So I think it was just the, just looking back at all the things I’ve done to help me feel better, but I really can’t pinpoint it, I think it was just a progression… because of COVID I got to do so much, like so much.” (Cali, 23 years)

Although the majority of youth participants identified gratitude during COVID-19, Kora, one of the younger participants, identified her difficulty finding something to be appreciative for during COVID-19. She stated:

“During COVID it was like kind of like it’s hard to find something that you really appreciate. ‘Cause like yeah there’s not really anything going on in your life and everything, oh yeah there’s like this crazy pandemic going on outside, that’s really it.” (Kora, 14 years)

### It’s OK to talk about it

This sub-theme of It’s OK to Talk About It refers to the participants’ acknowledgment and normalization of expressing emotions and feelings with others during COVID-19. Over the course of the pandemic, youth learned that asking for support was acceptable. This lesson was learned by Lexi, who stated:

“I think the biggest challenge for me personally was realizing that it’s okay to be vulnerable and it’s okay to have bad days and share that you’re having bad days with other people, like you don’t have to pretend to be perfect all the time, even when people say you know that you have it all figured out, like its okay not to, like you don’t have to like live up to those expectations or fulfill them. It’s okay to make mistakes and need support.” (Lexi, 23 years)

Participants spoke more freely to others about their own emotions, which contributed to their personal growth. This can be seen through Hana’s statement:

“I’ve gotten way more open about sharing that kind of stuff now… I guess especially because everything kind of blew up and now we live somewhere else, apart from like it’s much more easy, we’re like yeah, this is why we live somewhere else, hey do you want to hear what happened in like the past 17 years?” (Hana, 17 years)

Likewise, this was a similar lesson also learned by Lexi, who said:

“I think that that’s something that I’ve learned, is that there’s like so much power in talking to people and getting community support and you don’t have to go through anything alone. Yeah, so I think that that was a big one for me is just your mental health journey does not mean you have to navigate it on your own. it’s really about finding places and people that help you.” (Lexi, 23 years)

Recognizing the challenges of coping alone, Theo explained how the COVID-19 pandemic taught him the importance of social connectedness in supporting his mental health:

“I think when I, when I went back home and I realized like how much you know I was missing out on. That’s when it hit hard, like that it’s not, it’s not entirely possible to try to you know cope alone with this thing ‘cause I was trying to cope alone and that’s, that’s not possible, you can’t do that.” (Theo, 22 years)

Youth often discussed the benefits of connecting with oneself during the pandemic, allowing for introspection, meditation, and problem solving. Theo discussed journalling to help with his mental health:

“It was mostly like entry journals. Of like my mindset, my like mind state during those times. So, I would just, if I’m feeling some type of way I express it in writing. Sometimes I did write poetry. But it was supposed to actually be just so I could have an outlet to express myself.” (Theo, 22 years)

Cali shared her experience of self-talk while being active in nature:

“It really helped me mentally because you know how people listen to music when they go on walks? I used to bring my headphones with me but never used to listen to music. It was like more so with my thoughts being able to speak them out loud, ‘cause no one’s around me, to be able to jumble the mess that’s up here into like categories and to listen to like the wind, the leaves.” (Cali, 23 years)

In her second interview, Cali expanded on this strategy:

“Like I really did ask myself those uncomfortable questions that a lot of people don’t like asking. Like you know, what do I actually like? Am I able to do these things? I sat with those uncomfortable feelings until those uncomfortable feelings made me feel comfortable and that was a motto I kind of like lived by in 2022.” (Cali, 23 years)

Youth also relied on the informal mental health support of friends, peers and family members. Lexi explained her preference for support from her peers:

“I don’t know that like one-on-one therapy was necessarily like what I was looking for, I think I was just like craving more of these like deep and meaningful connections with like my peers.” (Lexi, 23 years)

Hana felt supported by family friends:

“I met these two people, they’re really good family friends, and I felt really comfortable with them, so I sort of like just like, oh you know this happened and they’re like, what the hell, and then I was just like, and I felt comfortable enough around them to like actually start talking about it. And then when like, they didn’t do anything bad, and they just tried to like help me, and I was just like, hi you know like maybe, I can just say shit and like you won’t get like angry with me for saying that shit? Well maybe I could try saying that shit ‘cause that can feel good.” (Hana, 17 years)

For other youth participants, sharing feelings and emotions with family was preferred and well established. Sienna explained comfort with confiding in her mother, “I think it was mostly my mom ‘cause it’s always been us… Like it’s, just been us, like girl power kind of thing” (Sienna, 20 years). As well, Yuki welcomed the support provided by an aunt to disclose his difficulties:

“I did have a supportive aunt who helped me through it. She sometimes would take me out and just have a nice day chatting to each other. I think that’s a really, she really helped me through the, she really helped me to, she gave me a little bit of a, of an escape, yes.” (Yuki, 19 years)

Participants additionally relied on formal supports to cope with their mental health.

For Sienna, having weekly time with a therapist was beneficial:

“I think just knowing that I had support every week was good too like and a safe space to talk things out. ‘Cause you know like my mom’s nice and all but you know she, she’s a not a therapist and it’s not fair to put that on her. And so having a safe space is, it was good.” (Sienna, 20 years)

Amirah, who also received therapy, explained, “especially now since I am like in therapy regularly and I feel like I’m able to, I know how to seek out professional help a little better, which I didn’t really know how to do that in the past” (Amirah, 22 years).

Youth participants also utilized phone and e-health services for mental health support, appreciating the confidentiality afforded by these services. Hana found these services helpful when she did not feel comfortable confiding with her family, “if you don’t have anybody, you can just call them, if you don’t want to like share stuff with your family because like, I’ll be honest like during the pandemic like, my parents were fighting and stuff, so I don’t think I wanted to talk to them a lot” (Hana, 17 years).

### Lessons in action

Lessons in action are the shared concepts youth viewed as important to have learned and incorporated into their practices following the COVID-19 pandemic. This sub-theme contributes to the theme of personal growth as it demonstrates what inspired development among youth in response to going through the COVID-19 pandemic. The opportunity to create a better mindset was the motivation Cali needed to create her unique plan, she stated:

“Reinvest in good things, you know, I just think that having this like negative mindset was very exhausting for those two years that I just don’t even want to go to that place anymore, I just don’t have it in me to be sad 24/7. I don’t want that for my life anymore, and so I’m trying to invest in me.” (Cali, 23 years)

Youth understood the importance of looking within themselves and developing strategies to self-manage one’s emotions. This is described by Mina, who noted, “I’m feeling that like I’m a calmer person. After all that, ‘cause I’ve learned to like interact with my family, interact with myself, and interact with my emotions” (Mina, 16 years). Lexi also reflected on a similar process, “it was like a little bit overwhelming at first to be kind of like alone with your thoughts, but then I realized like how important that is and how I’ve been neglecting it” (Lexi, 23 years).

The importance of social connection was also learned by youth during the COVID-19 pandemic. This importance was described by Imari, who noted, “we are dependent on each other and every person need help and emotional support, so that’s why the bonds become more stronger” (Imari, 24 years). Sienna identified the importance of human connection, stating:

“Just more grateful for like people too, realizing how much other people matter. And you can’t be alone, like humans need connection, I think that was one of the biggest things we learned from the pandemic is that inherently like humans need connections.” (Sienna, 20 years)

In dealing with daily life, youth had to come up with strategies to cope with demands. They found that COVID-19 taught them to prioritize the competing demands in their lives, for example, a busy work and school schedule, as noted by Jule:

“I guess going back to the positive, [COVID-19] just helped me prioritize things in life and like started learning a lot and do this intensive learning that I now do almost vitally.” (Jule, 23 years)

Mei also identified how COVID-19 resulted in understanding what her priorities were. She shared:

“If I’ve learned anything through this whole pandemic is we don’t know what the future’s going to hold in like a week, two weeks, a month, a year… it’s definitely made me realize what my priorities are.” (Mei, 22 years)

Throughout the pandemic, participants explained that they had to learn to be adaptable to the evolving situation. As Paige emphasized, “today that’s something that’s still like kind of relevant in everybody’s lives, like is just being able to like adapt to things and like figuring out how to make those things for you” (Paige, 14 years).

Participants were intentional in terms of organizing their plans and goals for specific outcomes during and after COVID-19. Jule explained:

“It’s very intentional the way I try to approach this stuff, like I know that if I start doing stuff like this mindlessly, especially socializing with people mindlessly, there’s something very wrong. That’s a red flag for me. That just tells me like okay I’m overextending myself and it’s only going to go downhill and symptoms are going to get worse ‘cause I’m so out of it. So I really have to sort of keep the reins in hand there.” (Jule, 23 years)

Participants further learned the importance of making time for themselves to maintain and improve their mental health and well-being during and after COVID-19. Self-care was described as a beneficial activity that made youth “feel good” (Theo, 22 years). For Cali, engaging in skincare was one strategy of self care that she used, noting, “I really started really taking care of myself and my skin, my skincare became a really big deal to me” (Cali, 23 years).

Another activity that the youth participants learned to prioritize for themselves during the pandemic was relaxation. Ruvi discussed her use of relaxation to cope, “And I used to feel yes, I’m breathing and I’m alive and yeah that’s, that’s all, I don’t have to do always, always I don’t have to run, I can, I can take a moment for myself and that will still be me and that’s fine” (Ruvi, 22 years). Youth viewed relaxation as a high priority item for themselves during and after COVID-19, with Amirah stating, “I think just really giving myself time to relax is like absolutely essential” (Amirah, 22 years).

Embracing nature was commonly viewed by the participants as a relaxing activity, allowing youth to connect, feel peaceful, and take a break. This coping strategy carried through to the present day. As described by Mina, “sometimes it feels stressful to like always be around people, and nature just like gives me a break and a chance to like, be myself and not having to prove anything” (Mina, 16 years). For Ruvi, she vividly recalled a moment in nature earlier in the day:

“This morning at 5 a.m. and we saw the sunrise and the trees they were looking so beautiful when they, like when the light fell upon them… It was looking like fall when trees get colorful, it was looking like that, and I was feeling wow, this is the moment I live for.” (Ruvi, 22 years)

### Art as a Window to the Past

Participating in the various arts-based activities throughout this study provided an opportunity for youth to reflect on their experiences with the COVID-19 pandemic. Many emphasized the benefits of participating, both personally and in research. As Cali explained:

“I think the way the method used like art-based, I think it’s really important in the type of research you’re doing ‘cause how would you collect this information in a survey? No, peoples’ experiences are more than just a question. So I think doing this was really helpful in reflection ‘cause you could place things that you wanted, you know like in a survey you can’t map out someone’s timeline you know, but in a picture you can. Which I think it was really, really helpful, I think it’s the best way.” (Cali, 23 years)

For Kora, she explained that her arts-based activity aided in her reflection on her COVID experience. She stated, “it definitely made me think about like things that happened during that time. ‘Cause it’s definitely not anything I think about like in my day to day” (Kora, 14 years).

For Ellis, reflecting on their past experiences through their journey map evoked complex emotions that, in the past, they tried to suppress, noting:

“It made me really sad doing it just because I’m also good at not thinking about things that make me upset so that I never have to deal with them. But I don’t know kind of getting it all out there and then I think leaving it there was, was nice… I think it also shows how far I’ve come.” (Ellis, 23 years)

## Discussion

Studies investigating the mental health impact of the COVID-19 pandemic among youth have predominantly been carried out using quantitative methods [52–54]. Similarly, other studies in this area have also been largely based on quantitative research [55–58]. This qualitative study described the experiences of Canadian youth throughout their journeys with the COVID-19 pandemic. To the best of our knowledge, this research study provides the first known qualitative, arts-based study exploring the experiences of Canadian youth and their mental health throughout all stages of the COVID-19 pandemic. This study is also the first known qualitative study to incorporate youth-informed guidelines for youth mental health and arts-based research in virtual environments [25].

The first theme, Entering COVID-19: Chaos, saw youth reflect on the initial stages of the COVID-19 pandemic. They noted initial feelings of confusion, recalled numerous changes to their day-to-day routines, perceived time differently, and experienced challenging emotions. In the second theme, During COVID-19: Introspection, youth took the time to reflect inwards upon themselves to understand their mental health and well-being during the peak of the COVID-19 pandemic and related restrictions. They learned to accept their pandemic reality in different ways and highlighted the coping strategies they employed, while also recalling the emotional toll this phase had on them. In the final theme, After COVID-19: Personal growth, youth described their experiences of personal growth as the urgency of the COVID-19 pandemic eased, including an increased sense of confidence, newfound gratitude, and an openness to sharing their emotions. Going through the pandemic also taught youth some lessons that applied to their current daily life.

Youth in this study echoed existing research of youth’s challenges during the early phases of the COVID-19 pandemic. Youth emphasized general hardships [59], uncertainty, change [60], and social isolation [61]. Emotionally, participants in this study similarly also described feelings of worry, anxiety [60,62,63], loneliness [61], and depression [64] that was heightened at the beginning of the pandemic and throughout the repeated lockdowns. This was amplified for youth with existing mental or physical health challenges [59,64].

In the current study, some participants described thriving and enjoyed better mental health and well-being in the new, more socially isolated environments of the early stages of the pandemic. This may be the result of individual personalities, as well as increased time and energy to pursue non-academic interests and learn new hobbies [61,65,66]. For some youth, they flourished under the fewer social obligations and pressures that occurred during these stages of the pandemic [67], and took the opportunity to rest and recharge [66].

During the pandemic, most youth in this study engaged in self-discovery activities. As a result of having additional time and opportunities for self-reflection, youth were able to learn about themselves and re-evaluate their values [60,64,68,69]. They were also able to challenge their capabilities and explore new interests and hobbies [59,60,63,70]. Like most of our participants, youth found success in their selected self-discovery activities and learned to adapt to their new lives with COVID-19 [66,71]. They worked to reframe their perspectives and challenges [63,69], as well as their sense of self [68]. Eventually, COVID-19 became a more positive experience [72,73].

Several youth highlighted challenges associated with the pandemic’s effect on their life course and its impact on the typical development of youth. During adolescence and early adulthood, typical milestones youth experience include completing education, finding employment and careers, forming relationships, learning to be independent, and establishing autonomy towards future goals [74,75]. These social and educational conditions aid youth in conceptualizing their futures, helping youth acquire the skills to link their present decisions to their later consequences and aspirations [75]. However, for several youth participants, the social and educational disruptions brought on by the pandemic impacted some of these typical transitions of young adulthood. Participants described changes to their career plans, as well as delays in immigration. Such disruptions to youth’s life course and milestone attainment can have implications for their development [14,76,77], including long-term risks to their mental health and well-being and lower academic achievement and post-secondary attendance [76].

Other youth also described feeling a pause in their life and a loss of time during their youth years as a result of pandemic restrictions and lockdowns. This experience of time has been noted in the literature as paradoxical due to the contradictory, yet simultaneous, feelings of time slowing down or pausing, as well as time accelerating [78]. Losing important life moments and age-specific celebrations associated with youth, as well as the concept of a continuous present – a lack of any change of scenery from the day-to-day – brought about loneliness and feelings of emptiness among several participant youth [78,79].

For youth in this study who were able to translate the pandemic’s challenges into successes, they achieved a more positive outlook of themselves and their future, in comparison to the few youth who experienced more difficulty attributed to the COVID-19 pandemic. Resilience may play a key role in mitigating COVID-19 pandemic-related trauma among youth [77,80]; hope and resilience in youth can also protect against COVID-19-related fear [81]. Youth in this study who did struggle with coping with the pandemic’s reality often had mental and/or physical health challenges, which may have influenced their experiences of the pandemic, as resilience is linked to mental well-being during the pandemic [82].

Coping strategies are considered an important personal resource for effective stress reduction, serving as mechanisms through which individuals are able to regulate their responses to stressful life circumstances within the pandemic [83]. While youth in this study highlighted a range of coping strategies (e.g., journalling, skincare, spending time in nature), a key insight from participants was the process through which they engaged in introspection to identify the most effective coping methods for themselves. Effective coping strategies can be highly individualized [84], and may require some level of trial and error for youth to identify the ones best suited to them. This is especially true during the unprecedented times of the pandemic, as many youth were forced to change their coping strategies due to pandemic restrictions [83]. This reflective approach demonstrates resilience among youth during periods of adversity. Notably, one coping strategy underexplored in the literature is the use of nature as a means of relaxation, a coping method that was frequently mentioned by participants in the study. Additionally, as youth participants moved through the different stages of the pandemic, they also explored different coping strategies to help them maintain their well-being, often engaging in trial and error to find the strategies that worked best for them at that stage. This variability in experiences with coping strategies highlights the importance of providing individualized mental health support for youth.

Having a greater connection with nature correlates with psychological well-being and an enhancement in overall quality of life [85,86]. Although research exploring nature as a coping strategy for youth mental health and well‑being is emerging, the body of work remains limited and warrants further inquiry. Existing studies indicate that time spent in natural environments is associated with positive mental‑health outcomes among young people, including feeling calmer, less anxious, and being more physically active [87]. In addition, during the pandemic, engagement with nature was shown to provide a respite, giving youth a sense of reassurance, safety and control to cope with their daily reality [88]. These positive mental-health outcomes have been attributed to nature’s ability to restore cognitive resources and reduce mental fatigue [89]. Further research is warranted, especially that which captures the nuanced experiences of youth as they utilize nature as a coping strategy.

In the current study, youth’s engagement in introspection often led to the development of greater confidence in themselves. This may be due, in part, to the self-discovery and coping activities they enacted to aide their well-being during the pandemic, as such activities can help youth gain greater insights about themselves [83,90]. The co-occurring passage of time and maturation of youth over the pandemic may also have played a role in their enhanced self-confidence, as self-esteem tends to increase in youth after age 15 [91,92].

As the initial emergent phases of the COVID-19 pandemic waned, youth in this study described rich experiences of positive personal growth. Similar to the literature, youth expressed gratitude for the family, friends, and support people (e.g., educational and health) in their lives [61,63,69,71,72,93]. Having social connection was a need that was recognized by several youth, which was often lacking during the pandemic. This has the potential to significantly impact youth’s mental health [94], as social connection, as opposed to social isolation, strengthens youth mental health and well-being [95,96].

For several youth participants, over the course of the pandemic, they expressed greater acceptance towards talking about their mental health with others and a greater willingness to ask for help when they needed it. Those who experienced higher levels of stress and worry for longer periods of time during the pandemic, tend to express a more positive attitude towards seeking help [97]. Pandemic-related fears and life course stressors can also lead youth to seek help [97,98]. Destigmatizing the conversation around mental health and related challenges can also encourage this, as help-seeking behaviours in adolescents can be influenced by personal and perceived mental health stigma, as well as individual attitudes and knowledge around help-seeking [99,100].

By applying guidelines for using ABM with youth in virtual environments [25], this strengthened youth’s ability to meaningfully and safely participate in the research. This study incorporated a wide range of arts-based activities that helped reveal the nuances of youth’s mental health throughout the pandemic that may not have been easily identified using traditional qualitative methods alone. Creative methodologies help youth reframe their perspectives and enhance their ability to see alternative viewpoints [25,68,90]. They can also enhance accessibility and yield new insights in research [101], as well as aid in sharing youth’s experiences when words alone do not suffice [31]. Through a variety of activities, including ecomaps, journey maps, and youth’s own choice of arts-based creations (e.g., poetry, mood boards, body maps, drawings, songs, etc.), this provided a means for research that sustains mindful presence. Such research maintains “careful thought, receptive attention, and awareness of what is taking place to provide space for participants’ authentic expression, ensure participants have meaningful research experiences, and enhance researchers’ understanding of participants’ perspectives” [25, p. 13].

As collaborative research has been identified as essential for understanding the long-term effects of COVID-19 for youth with mental health issues [62], the inclusion of youth co-researchers in this study provided youth perspectives throughout all stages of the research [67,102]. Having meaningful engagement with youth in these ways also promotes the uptake of research that is more culturally responsive and inclusive [36,37].

### Recommendations

Pulling from participants’ lived experiences and the literature, the following are three recommendations formed to support mental health and well-being among youth, particularly during future pandemics and health crises.

Provide youth with understandable, age-appropriate, and timely mental health information and support [56]. While participants appreciated the informal support of friends and family, utilizing formal supports, such as therapy and e-mental health services, were highlighted as particularly beneficial. Formal support should be private, non-judgmental, non-stigmatizing, safe, diverse, and inclusive. Youth should not be expected to navigate the many barriers associated with help-seeking alone [60].
Provide opportunities for youth to share their stories and reconstruct self-narratives to encourage resilience and improve their mental health and well-being. Participants learned, over the course of the pandemic, that it was okay to talk to others about their feelings and mental health challenges. By normalizing their experiences, feelings, and emotions, this may contribute to more positive mental health and well-being, even during crises [68]. This may be achieved through regular conversations between youth and youth caregivers and by using a range of arts-based activities [25]. Participants found the arts-based activities helpful to reflect on their experience and process the emotions they went through. Through these modalities, youth should be encouraged to share the ways they “embody, negotiate, and manage emotions” [90, p. 2]. In doing so, youth can gain a better understanding of their situation to make sense of it within their own context and help them develop greater control of their situation [66].
Provide young people with dedicated space for introspection, allowing them time to explore and engage in trial and error as they determine optimal coping methods for challenging situations. Introspection served an important role for several participants to help them process their experiences and feelings related to the COVID-19 pandemic. Since youth were forced to change many of their coping strategies as a result of the pandemic [83], giving youth the time to find new strategies is of key importance. Moreover, youth should be supported in their autonomous choice of coping strategies, including those that may fall outside conventional categories (for example, connecting with nature). Due to the individual differences in the habitual use and choice of coping strategies and their different psychological outcomes, it is a highly personal decision to make [22,83].

### Limitations and future research

In this study, 19 participants were recruited across Canada. Though the intention was to include the voices of youth from across Canada, the majority (n = 13) resided within the province of Manitoba. Future studies should seek to explore the experiences of youth from across the Canadian provinces, with focused recruitment targeting youth residing in rural and remote areas. Acknowledging the convenience sampling used for participant recruitment and the smaller sample size obtained, there is the potential that the findings may reflect the perspectives of youth already partial to engaging in and reflecting on their mental health and coping strategies. There was also greater representation of female youth voices, with fewer male and non-binary youth. The ages of the participants skewed towards older, with most (n = 15) being 19 years or older, which limited the ability to draw comparisons between and within younger and older youth. Moving forward, additional studies should be undertaken to continue the study of COVID-19’s impact on the mental health of young people long-term, with intention to include youth who are often underrepresented in this research. Further research should also explore how arts-based activities may facilitate introspection and reframing for more positive mental health and well-being experiences, particularly during times of crisis.

## Conclusion

Through the voices and arts-based activities of 19 youth from across Canada, their journeys through the COVID-19 pandemic and the resulting impact on their mental health experience was gleaned. Using qualitative and ABM, youth were able to recount key time points through the pandemic, including the chaos of entering the pandemic, a time of introspection during, and their resulting personal growth after. Supported by guidelines co-developed by youth for using ABM in virtual environments, this provided a more complete picture of these key points from youth’s perspectives, and ensured a safe space for youth to creatively reflect on and share their experiences. Recommendations include increasing youth mental health support and guidance, as well as incorporating ABM to encourage introspection and help facilitate coping strategies. ABM provide a unique means to understand youth’s experiences, while strengthening their ability to participate in the research safely and meaningfully [25].
